# Discrete Changes in Glucose Metabolism Define Aging

**DOI:** 10.1038/s41598-019-46749-w

**Published:** 2019-07-17

**Authors:** Silvia Ravera, Marina Podestà, Federica Sabatini, Monica Dagnino, Daniela Cilloni, Samuele Fiorini, Annalisa Barla, Francesco Frassoni

**Affiliations:** 1 UOC Laboratorio Cellule Staminali post-natali e Terapie Cellulari, IRCCS Istituto Giannina Gaslini, Genoa, Italy; 2 Department of Experimental Medicine, University of Genoa, Genoa, Italy; 3 Department of Clinical and Biological Sciences, University of Turin, Turin, Italy; 4 Department of Informatics, Bioengineering, Robotics and Systems Engineering, University of Genoa, Genoa, Italy; 5 Department of Mathematics, University of Genoa, Genoa, Italy

**Keywords:** Predictive markers, Biogeochemistry

## Abstract

Aging is a physiological process in which multifactorial processes determine a progressive decline. Several alterations contribute to the aging process, including telomere shortening, oxidative stress, deregulated autophagy and epigenetic modifications. In some cases, these alterations are so linked with the aging process that it is possible predict the age of a person on the basis of the modification of one specific pathway, as proposed by Horwath and his aging clock based on DNA methylation. Because the energy metabolism changes are involved in the aging process, in this work, we propose a new aging clock based on the modifications of glucose catabolism. The biochemical analyses were performed on mononuclear cells isolated from peripheral blood, obtained from a healthy population with an age between 5 and 106 years. In particular, we have evaluated the oxidative phosphorylation function and efficiency, the ATP/AMP ratio, the lactate dehydrogenase activity and the malondialdehyde content. Further, based on these biochemical markers, we developed a machine learning-based mathematical model able to predict the age of an individual with a mean absolute error of approximately 9.7 years. This mathematical model represents a new non-invasive tool to evaluate and define the age of individuals and could be used to evaluate the effects of drugs or other treatments on the early aging or the rejuvenation.

## Introduction

Aging is a multifactorial process characterized by a progressive decline of physiological functions^1^, that determines an increment of vulnerability and the relative risk of developing diseases, i.e. cancer, cardiovascular disorders, diabetes, neurodegeneration and death^2,3^. Several alterations are involved in the aging process, including deregulated autophagy, mitochondrial dysfunction, telomere shortening, oxidative stress, systemic inflammation, and metabolism dysfunctions^2,4^. Recently, also an involvement of epigenetic modifications has been proposed^5^ as cause of aging, allowing to develop an “aging clock” based on the degree of DNA methylation, which increases with the age^6^. However, for several years, aging has been considered the result of damages accumulation due to an excessive production of reactive oxygen species (ROS), leading to the formulation of the “Mitochondrial Theory of Aging”^7,8^. This theory is based on the concept that mitochondria are one of the main sources of oxidative stress^9–11^ and that mitochondrial DNA (mtDNA) displays a great rate of mutation together with a less efficient repair machinery, in comparison to nuclear DNA (nDNA)^12^. Once a mtDNA mutation threshold has been reached, irreversible oxidative damage is formed, which leads to dysfunction of mitochondrial metabolism^13^, accelerating the oxidative stress production^14^.

Therefore, in this work, we evaluated the changes of energy metabolism in mononuclear cells (MNC) isolated from peripheral blood (PB), obtained from healthy population with an age between 5 and 106 years, analysing the oxidative phosphorylation (OXPHOS) function and efficiency, the ATP/AMP ratio, the lactate dehydrogenase (LDH) activity and malondialdehyde (MDA) content, to propose a mathematical model based on Machine Learning (ML) that, once trained on the collected data, aims at predicting the age of an individual on the basis on these metabolic markers.

ML is the branch of computer science that aims at inferring the input-output relationship underlying a collection of examples^15^. In order to devise such relationship, ML models need to be *trained* on a set of input-output pairs, where the input data *x* are described as *d*-dimensional vectors and *y* is the output variable. In particular, predicting a real-valued output poses a *regression* problem. The final goal of ML is to devise predictive models that, once trained on a first dataset, will perform well on new, and previously *unseen*, samples, *i*.*e*. the so-called test set. A ML model that achieves accurate predictions *only* on the training set is said to be *over-fitting*. This is an unwanted behaviour and precautions must be taken in order to avoid it. The most adopted technique to prevent over-fitting is called *regularization* and it consists in introducing additional information that, reducing the space of the possible solutions, helps the model to achieve good generalization properties^15^. In particular, *sparsity*-enforcing methods are one of the most relevant regularization strategies for biological applications. Sparse models identify subsets of variables upon which the predictive model is based. This subset is identified as the set of variables having non-zero weights. The remaining variables have zero weight and therefore are discarded as not relevant.

## Results

All the experiments were carried out on MNC isolated from PB obtained from the healthy population with age between 5 and 106 years. The distribution of the population was reported in Supplementary Fig. 1, and the information about the human subjects (i.e.: gender and age), as well as the value of the evaluated biochemical markers are reported in Supplementary Dataset.

### The energetic status and the mitochondrial aerobic metabolism changes in MNC, during the physiological aging

**Figure 1:**
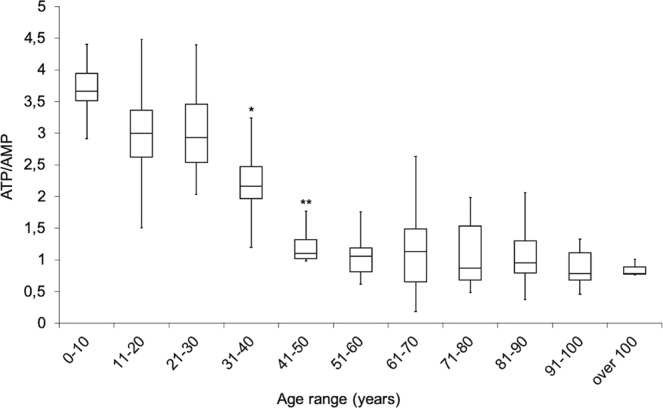
Changes of MNC energy status during aging. Graph shows the ATP/AMP ratio, as a marker of the energy status of MNC isolated from peripheral blood (PB), obtained from healthy population with an age between 5 and 106 years. The population is divided by decades. The values decrease progressively with aging. * or ** indicate, respectively, a significant difference for p < 0.05 or p < 0.01 between the marked decade and the previous decade.

To determine the energy status of MNC, the intracellular concentration of ATP and AMP was evaluated, and the ratio ATP/AMP was calculated (Fig. 1, Panel A). Data shows that the energy status changed with the age, decreasing progressively. More in details: values did not change in the first three decades of age (0–30 years), showed a modest decrement between 30 and 40 years of age (p < 0.05 vs decade of 0–30 years of age), drastically decreased between 40 and 50 years (p < 0.01 vs the first four decades) and remained low, but stable, in the subsequent decades. Moreover, analysing the single data of ATP and AMP cellular concentration (Supplementary Fig. 2), it is evident that the cause of the decrement of ATP/AMP ratio was principally the increment of AMP concentration during the aging, in particular in the decades of 31–40, 41–50 and 51–60 years (p < 0.01 vs the previous decade).

**Figure 2:**
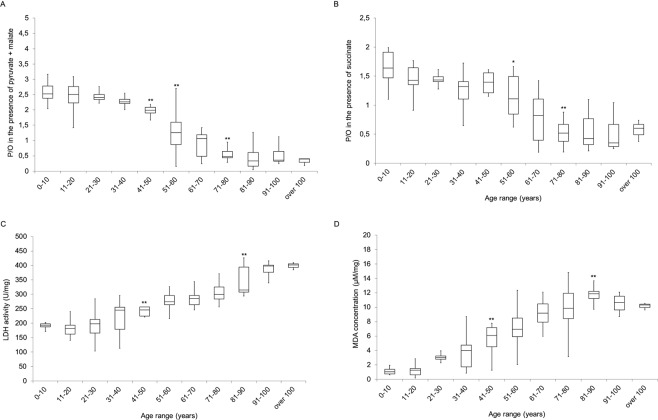
Changes of MNC glucose metabolism during aging. All data are obtained using MNC isolated from peripheral blood (PB) of healthy subjects with an age between 5 and 106 years. (**A**,**B)** Show the efficiency of the oxidative phosphorylation, expressed as P/O ratio, in the presence of pyruvate + malate or succinate, respectively. In both cases, the values decrease during aging, suggesting an uncoupling between oxygen consumption and ATP synthesis, which determines a low efficiency in energy production. (**C)** Reports the lactate dehydrogenase activity, as a marker of anaerobic glucose metabolism. The values increase in the elderly subjects, indicating that the lactate fermentation enhances parallel to the aging. (**D)** Shows the level of malondialdehyde (MDA), as a marker of oxidative stress, which increases with aging, probably due to the minor OXPHOS efficiency. In each panel, the population is divided by decades. * or ** indicate, respectively, a significant difference for p < 0.05 or p < 0.01 between the marked decades and the previous decade.

Further on, the efficiency of oxidative phosphorylation (OXPHOS) was evaluated in term of P/O value, calculated as the ratio between ATP synthesis (ATP-synth) and oxygen consumption rate (OCR). These parameters were evaluated in the presence of pyruvate + malate (Pyr + Mal) or succinate to stimulate the pathways formed by complexes I, III and IV and complexes II, III and IV, respectively. The P/O ratio after Pyr + Mal stimulation followed a similar trend observed for the ATP/AMP ratio (Fig. 2, Panel A). In particular, in the first four decades of age (0–40), the P/O value was around 2.5, which corresponds to the reference values^16^, while it showed a slight decrement in the range between 41 and 50 years (p < 0.01 vs the first three decades) and decreased drastically from 51 years onwards (p < 0.01 vs the first three decades). A further decrease was observed in the 71–80 decade (p < 0.01 vs the previous decades), then the P/O remained stable, but low, after the 80 years of age. Also the P/O ratio calculated after succinate induction decreased with the age, but its impairment was less evident around the fourth and fifth decades, appeared more marked only after 60 years (p < 0.05 compared with the younger decades) and definitely low around in the eighth decades (p < 0.01 vs the previous decade) (Fig. 1, Panel C).

Considering that the loss of mitochondria efficiency could determine the enhancement of the anaerobic metabolism, the activity of lactate dehydrogenase (LDH) has been evaluated as a marker of the anaerobic glycolytic pathway. Data show an increment of LDH activity in MNC with aging: the values were similar in the first four decades, increasing significantly in the fifth decade (p < 0.05 vs the previous decade) and reached the maximum after 81 years (p < 0.01 with respect the previous decade) (Fig. 2, Panel C).

Moreover, since the uncoupled OXPHOS metabolism is often associated with an increment in the oxidative stress production^11^, which induces damages on proteins, nucleic acid and membrane, the level of malondialdehyde (MDA), a marker of lipid peroxidation, has been evaluated in MNC (Fig. 2, Panel D). MDA concentration remained stable until the 20 years of age, increased around the third decade, became significant different after the 41 years of age with respect to the younger decades (p < 0.01 vs the younger decades) and reached the maximum level around the ninth decade (p < 0.01 vs the younger decades). Interestingly, after the 80 years of age, MDA values remained stable, probably due to the physiological metabolic slowdown.

### Specific metabolic fingerprint characterizes each age

**Figure 3:**
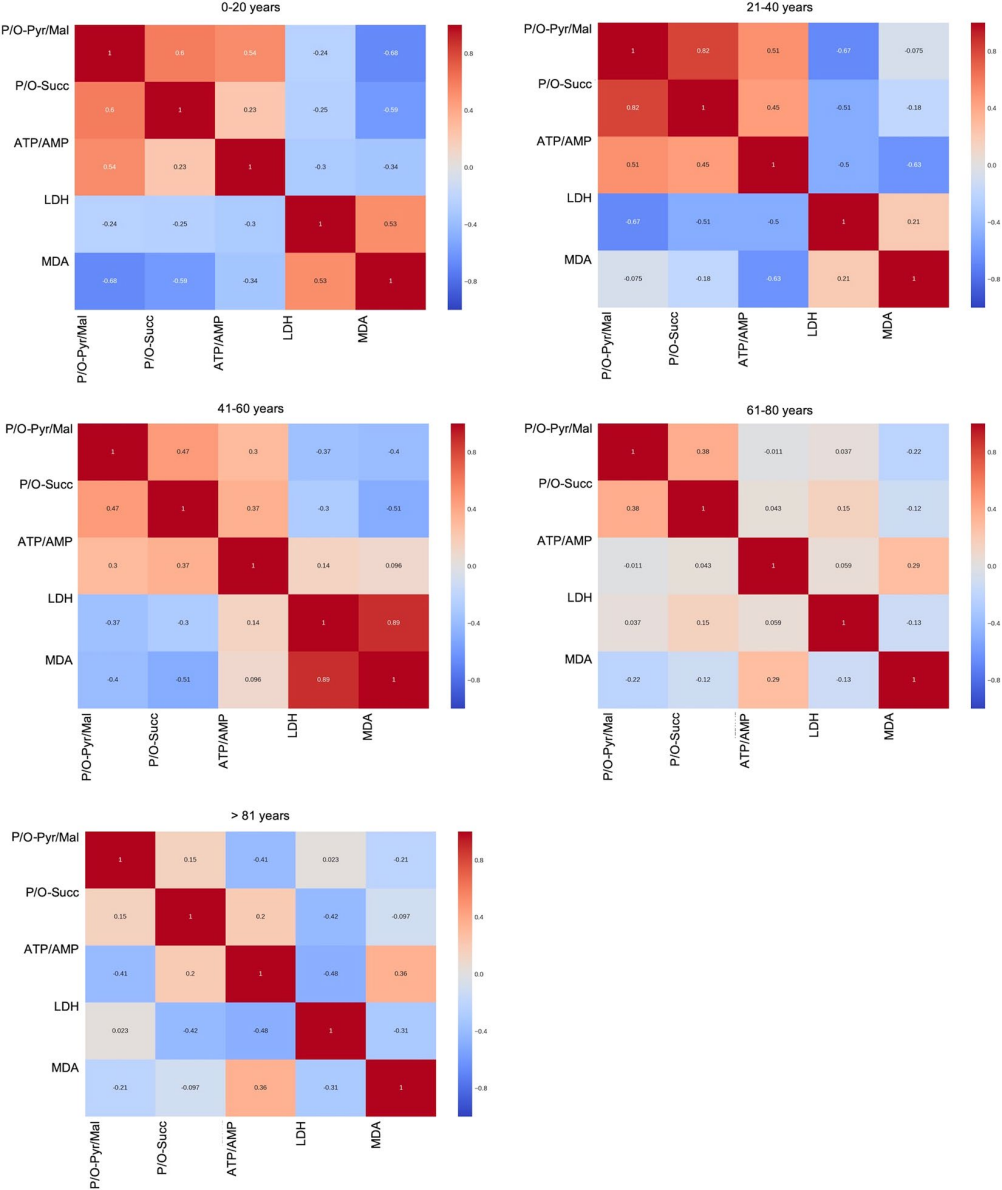
Changes of correlation among the biochemical markers during aging. Heatmaps reported in this figure show the correlations among the biochemical markers reported in Figs 1 and 2. Population is divided into 5 groups, one for each two decades (0–20, 21–40, 41–60, 61–80, >81). Dark red entries indicate a strong positive correlation, white entries represent no correlation and dark blue entries correspond to strong negative correlation. Each age group is characterized by a specific pattern of correlations.

In this work, we performed exploratory data analysis estimating the collinearities among measured variables via pairwise Pearson Correlation Coefficient (*r*) (see Eq. 3 in Materials and Methods section). Evaluating *r* for each pair of markers results in a symmetric heatmap, in which dark red entries are associated with strong positive correlation, white squares represent no correlation and dark blue squares correspond to strong negative correlation, see for example one of the panels in Fig. 3. Dividing the collected data into 5 groups, one for each two decades (0–20, 21–40, 41–60, 61–80, >81), we obtained 5 heatmaps, which represent the correlations among different metabolic parameters during aging (Fig. 3). In particular, it is possible to observe that each age group is defined by a specific pattern of correlations. For example, the younger samples (0–20 years of age), characterized by an efficient aerobic metabolism, display a positive correlation with P/O values and ATP/AMP ratio and a negative correlation between P/O values and both LDH activity and MDA levels. Conversely, in the old samples (>61 years of age), P/O values negatively correlated with the ATP/AMP ratio, while they did not significantly correlate with LDH activity.

Moreover, also the correlation between ATP/AMP ratio and MDA production changes with the age, appearing negligible for the younger samples (<21 years of age), negative for people of age between 20 and 60 years of age and positive for samples over 60 years of age.

### A mathematical model predicts the age based on the energy metabolism markers

The proposed predictive model, *e*.*g*.*:* the “biochemical aging clock”, is achieved by posing a linear age-regression problem from the set of the following biochemical biomarkers (measured in MNC): ATP, AMP, MDA, LDH, ATP/AMP ratio, P/O-Pyr + Mal, OCR-Pyr + Mal, ATP synth-Pyr + Mal, P/O-Succinate, OCR-Succinate and ATP synth-Succinate.

We relied on one of the most popular sparse regression methods that is the Elastic-Net^17^. The Elastic-Net solution can be achieved by solving the minimization problem stated as follows (Eq. 1):

1 $$\hat{w}=argmi{n}_{w\in {{\mathbb{R}}}^{d}}\frac{1}{n}{({y}_{i}-w{x}_{i})}^{2}+\alpha [\lambda |w|+(1-\lambda ){w}_{2}^{2}]$$

The first term of the functional is called *loss function* and it measures the adherence between the actual output and the predictions in terms of average squared Euclidean norm. The second term, in square brackets, is the regularization penalty. The Elastic-Net model adopts a mixed penalty that achieves smooth and sparse solutions, where the correlation among the input variables is preserved and can be tuned to simultaneously control the smoothness and the sparsity of the achieved solution. In particular, when **λ** = 1 the effect of the L2-norm vanishes and the regularization term enforces a level of sparsity in the solution which is proportional to, as in the Lasso^18^. On the other hand, when **λ **= 0 the effect of the L1-norm vanishes and the regularization parameter ***α*** controls the smoothness in the solution, as in Ridge regression^15^. Balancing the trade-off between L2 and L1 is the key to obtain accurate and interpretable solutions.

Thanks to the sparsity-enforcing properties of the Elastic-Net penalty, we were able to identify a reduced number of relevant biomarker measures. In fact, out of the 11 variables, the model automatically selected only the 8 most predictive measures (*i*.*e*.*:* OCR-Succinate, P/O-Pyr + Mal, PO-Succinate, ATP synth-Pyr + Mal, AMP, MDA, LDH, ATP/AMP), which correspond to the nonzero elements. By contrast, 3 null elements of *w* correspond to the discarded variables (ATP, OCR-Pyr + Mal, ATP synth-Succinate).

Therefore, the final model obtained after the final refit on the training set assumes the following form (Eq. 2):

2 $$\begin{array}{lll}predicted\,age & = & 0.01\cdot OCR \mbox{-} Succinate-7.30\cdot P/O \mbox{-} Pyr+Mal\\  &  & -0.31\cdot P/O \mbox{-} Succinate\\  &  & -0.01\cdot ATP\,synth \mbox{-} Pyr+Mal\\  &  & +10.29\cdot AMP+2.05\cdot MDA\\  &  & +\,0.01\cdot LDH-4.22\cdot ATP/AMP+49.21\end{array}$$

This model, evaluated on the test set, achieves an absolute error of 7.9 years explaining the 84.3% of the variance. In order to better understand the model behaviour, we can see it working in practice. Table 1 shows relevant values of the molecular biomarkers corresponding to 14 subjects randomly extracted from the test set. This ensures that the weights of the proposed “biochemical aging clock” (Eq. 2) are estimated without taking into account such measures, hence preventing overfitting. The last columns on the right of Table 1 show real and predicted ages, and their unsigned difference.

**Table 1:** Example of Eq. 2 application to predict age on the basis of glucose metabolism markers.

Sample ID	Biochemical markers								predicted age (years)	real age (years)	abs (error) (years)
	*OCR-Succinate*	*P/O-Pyr* + *Mal*	*P/O-Succinate*	*ATP-Pyr* + *Mal*	*AMP*	*MDA*	*LDH*	*ATP/AMP*			
*#165/2017*	12.24	2.69	1.38	29.93	0.57	3.73	205.98	2.49	34	30	+4
*#228/2017*	11.35	2.36	1.34	31.26	0.66	4.21	241.56	2.19	40	35	+5
*#416/2016*	12.63	0.96	1.19	15.67	0.56	6.95	326.47	1.87	57	54	+3
*#91/2017*	12.74	0.86	0.62	11.75	0.56	5.46	265.11	1.11	58	56	+2
*#81/2017*	10.45	1.28	0.88	14.4	0.65	6.53	301.27	0.79	59	55	+4
*#418/2016*	19.31	1.03	0.87	23.65	0.53	8.29	345.19	1.5	61	66	+5
*#83/2017*	16.21	0.86	0.98	11.02	0.72	5.99	265.78	0.61	62	57	+5
*#509/2016*	23.28	1.36	0.48	16.69	0.79	9.41	251.36	0.68	66	65	+1
*#517/2016*	43.64	0.25	0.23	5.55	0.53	9.63	285.41	1.49	70	64	+6
*#76/2017*	17.12	1.06	1.07	14.22	0.82	9.2	302.75	0.44	70	69	+1
*#504/2016*	39.16	0.48	0.2	7.17	0.49	12.75	256.78	1.85	72	72	0
*#506/2016*	50.6	0.29	0.4	5.25	0.66	9.18	298.75	0.88	72	71	+1
*#75/2017*	9.87	0.73	0.21	11.43	0.8	10.99	382.89	0.39	77	83	−6
*#94/2017*	13.64	0.44	0.39	7.24	0.8	13.93	385.14	0.38	85	82	+3

The table represents an example of Eq. 2 application on 14 subjects randomly extracted from the test set. For each subject, the biochemical markers of glucose metabolism, the real and predicted ages and their difference are reported.

## Discussion

In this work, we described a new aging-clock, based on the changes of glucose catabolism in the aging, able to predict the age of an individual. The biochemical markers have been evaluated on MNC, which have been chosen because blood cells are considered an excellent model to evaluate the metabolic status of the entire organism^19^. Moreover, it was also shown that mutations identified in the peripheral blood cells (PBC) of aged “normal subjects” are statistically correlated with degenerative diseases^20^.

The idea of our work is based on the energy metabolism changes that occur with aging. In fact, as shown by our results on MNC, the glucose metabolism shifts from aerobic to anaerobic with the age, influencing the cellular energy status. In particular, our data show that, during aging, the oxygen consumption is not completely coupled with the ATP synthesis, determining a reduction of ATP availability (Fig. 1 and Supplementary Fig. 1) and making more difficult the AMP recycling through the enzymes that regulate the energy balance such as adenylate kinase^21,22^. Moreover, the uncoupling status of OXPHOS machinery determines an increment of ROS production, as shown by the high MDA level observed after the sixtieth and later decades (Fig. 2, Panel C), triggering a vicious circle, in which the damage of the mitochondrial inner membrane induces an increment of oxidative stress and the relative structural failures. An attempt to restore the ATP level is represented by the increment of LDH activity (Fig. 2, Panel D), probably to convert the NADH to NAD^+^, to restore the correct pool of oxidized coenzyme. However, it is important to note that LDH increment does not represent a “choice” for the cell, but the unique alternative to produce ATP from glucose.

All these data are confirmed by the heatmaps, which display a correlation among the evaluated biochemical markers in the different decades (Fig. 3). In fact, the positive correlation within P/O ratio and ATP/AMP ratio and the negative correlation between P/O ratio and LDH activity or MDA levels are typical of the samples of younger subjects (0–20 years of age), in which a perfect coupling between oxygen consumption and ATP synthesis occurred. By contrast, in the samples of older individuals, P/O values negatively correlated with the ATP/AMP ratio, and did not significantly correlate with LDH activity, confirming that, during aging, the energy production through OXPHOS becomes less efficient, inducing the increment of aerobic glycolysis to compensate, at least in part, the reduced production of ATP.

Interestingly, the trend of the correlation between P/O and ATP/AMP is more evident in the presence of pyruvate + malate with respect to that observed after succinate induction. This may depend by the fact that the pathway led by Complex I contributes more to the production of the proton gradient necessary for energy production through ATP synthase, with respect the Complex II pathway^23^. Moreover, the changes from negative to positive correlation between ATP/AMP ratio and MDA production changes with the age, confirms that MNC continue to use the oxidative phosphorylation as energy source, but this is associated with an increase in oxidative stress production, which could contribute to the progress of aging, determining a vicious circle.

Our data suggest that the critical period seems be around between the fifty and sixty decades; in fact, subsequently the energy metabolism continues to change, but more slowly, and stabilizes after 80 years of age. This could depend by the fact that over 60 years of age the level of oxidative stress is so high to become critical for the integrity of the cellular structure, determining a damage. In fact, it is known that mild inhibition of mitochondrial respiration was shown to extend lifespan in many species such as *C*. *elegans*, *Drosophila* and mice^24,25^. Therefore, it is possible to speculate that around the early phases of aging (30–40 years of age) the increment of oxidative stress production could be considered favoured to drive towards cell proliferation devoted to the renewal^26^. However, the increment of cellular proliferation, requiring energy, increases the mitochondria metabolism and the relative oxidative stress production, reaching a critical amount that determining the aging in the later decades^26^.

All these data were used to perform a sparse linear regression analysis, in order to evaluate whether the age of an individual can be predicted from biochemical markers and to identify which are the predictive ones.

The best prediction is achieved by the model described in Equation 2. The model is a linear combination of the metabolic markers, where only 8 out of 11 are associated to non-zero weights. The remaining 3 markers are discarded as they do not contribute to the best prediction. In particular, the weights are learned from the data via the Elastic-Net algorithm (Hastie *et al*. 2009) that automatically discards the irrelevant metabolic markers and, at the same time, takes into account the correlated ones, as shown in the heatmaps (Fig. 3).

The choice of this algorithm guarantees a compact, accurate and easily interpretable model, which is robust to the noise affecting the data. In order to test the predictive properties of this Elastic-Net-based procedure, we repeated the model identification procedure 100 times on 100 random re-sampling of the input dataset^27^, achieving a mean absolute error of 9.7 ± 1.3 years and a mean explained variance of 77 ± 7%.

In conclusion, the mathematical model proposed in this work represents a new non-invasive tool to evaluate and define the age of individuals, on the basis of biochemical/bioenergetic markers. This approach could help to evaluate the effects of drugs or other treatments on early aging or rejuvenation. For example, it is known that the survival rates of pediatric cancers have improved tremendously over the past four decades, but the therapy responsible for this survival can also produce adverse long-term health-related outcomes^28–30^. In particular, it was observed that the prevalence of frailty in young adult treaded in childhood for cancer is similar to that expected among adults > 65 years old from the general population^31^. Therefore, the possibility to predict the real age in childhood cancer survivors could be useful to act promptly preventing the damages due to the early aging.

## Methods

### Reagents

All chemicals were purchased from Sigma Aldrich (St. Louis, MO, USA), unless otherwise indicated. Ultrapure water (Milli-Q; Millipore, Billerica, MA, USA) was used throughout. All other reagents were of analytical grade.

### Samples

This study was performed on mononuclear cells (MNC) isolated from peripheral blood (PB), obtained from healthy population with age between 5 and 106 years. For the participants under the age of 18 years, we have obtained the informed consent from a parent and/or legal guardian. In total, we have collected 118 samples from a heterogeneous group of individuals, not affected by chronic diseases and with different life-styles. PB samples were transferred into the laboratory and analysed within 24 hours from collection. All participants provide their written informed consent to participate in this study. Exclusion criteria were positivity for HBV, HCV and HIV. For each subject, 7–10 ml of PB were employed.

This study was approved by the Regional Ethics Committee of Liguria (N° 096REG2014) and all the experiments were performed in accordance with relevant guidelines and regulations.

### Evaluation of ATP/AMP ratio

The ATP and AMP quantification was based on the enzyme coupling method^32^. 20 µg of total protein were used for both assays. Briefly, ATP was assayed following NADP reduction, at 340 nm. The medium contained: 50 mM Tris HCl pH 8.0, 1 mM NADP, 10 mM MgCl_2_, and 5 mM glucose in 1 ml final volume. Samples were analysed spectrophotometrically before and after the addition of 4 µg of purified hexokinase plus glucose-6-phosphate dehydrogenase. AMP was assayed following the NADH oxidation at 340 nm. The medium contained: 100 mM Tris-HCl pH 8.0, 75 mM KCl, 5 mM MgCl_2_, 0.2 mM ATP, 0.5 mM phosphoenolpyruvate, 0.2 mM NADH, 10 IU adenylate kinase, 25 IU pyruvate kinase, and 15 IU of lactate dehydrogenase.

### Oximetric analysis

The oxygen consumption was measured with an amperometric O_2_ electrode in a closed chamber, magnetically stirred, at 37 °C (Unisense, DK). For each assay, 200,000 cells were used. Sample was suspended in a medium containing: 137 mM NaCl, 5 mM KH_2_PO_4_, 5 mM KCl, 0.5 mM EDTA, 3 mM MgCl_2_ and 25 mM Tris–HCl, pH 7.4 and permeabilized with 0.03 mg/ml digitonin for 10 min. To stimulate the pathway composed by Complexes I, III and IV, 5 mM pyruvate plus 2.5 mM malate were added. To activate the pathway composed by Complexes II, III and IV 20 mM succinate was used^33^.

### Evaluation Fo-F1 ATP synthase activity

ATP synthesis was measured by the highly sensitive luciferin/luciferase method. Assays was conducted at 37 °C, over 2 min, by measuring formed ATP from added ADP. 200,000 cells were added to the incubation medium (0.1 ml final volume) containing: 10 mM Tris-HCl pH 7.4, 50 mM KCl, 1 mM EGTA, 2 mM EDTA, 5 mM KH_2_PO_4_, 2 mM MgCl_2_, 0.6 mM ouabain, 0.040 mg/ml ampicillin, 0.2 mM di-adenosine-5′penta-phosphate, 0.2 mM and the metabolic substrate: 5 mM pyruvate plus 2.5 mM malate or 20 mM succinate. The cells were equilibrated for 10 min at 37 °C, then ATP synthesis will be induced by addition of 0.2 mM ADP. The ATP synthesis was measured using the luciferin/luciferase ATP bioluminescence assay kit CLSII (Roche, Basel, Switzerland), on a Luminometer (GloMax® 20/20 Luminometer – Promega, Wisconsin, USA). ATP standard solutions (Roche, Basel, Switzerland) in the concentration range 10^−10^–10^−7^ M was used for calibration^33^.

### Lactate dehydrogenase assay

To assay the glycolytic flux, the activity of lactate dehydrogenase (LDH; EC 1.1.1.27) was measured at room temperature on 20 µg of MNC homogenate. The reaction mixtures contained: 100 mM Tris-HCl pH 7.4, 0.2 mM NADH and 5 mM pyruvate (Ravera *et al*. 2013). NADH molar extinction coefficient was considered 6.22 mM^−1^ cm^−1^, at 340 nm. Enzymatic activity was expressed as mU/mg of total protein (nmol/min/mg of protein)^34^.

### Evaluation of lipid peroxidation

To assess lipid peroxidation, the malondialdehyde (MDA) level was evaluated, using the thiobarbituric acid reactive substances (TBARS). The TBARS solution contained: 15% trichloroacetic acid (TCA) in 0.25 N HCl and 26 mM 2-Thiobarbituric acid. To evaluate the basal concentration of MDA, 600 µl of TBARS solution was added to 50 µg of total protein dissolved in 300 µl of milliQ water. The mix will be incubated for 40 min at 100 °C, then centrifuged at 14,000 rpm for 2 min and the supernatant was analysed spectrophotometrically, at 532 nm^35^.

### Statistical analysis

Biochemical data were analyzed by one-way ANOVA followed by Bonferroni post hoc test, using Sigma Stat software (Sigma Stat Software, Inc., San Jose, CA, USA). p < 0.05 was considered significant.

### Mathematical model development

In order to identify a mathematical model capable of predicting the metabolic age of an individual given the corresponding metabolic biomarkers, we rely on a three-step procedure. The first step consists in splitting the 118 collected samples in two non-overlapping sets, namely: training set (88 samples) and test set (30 samples). Cardinality of training and test sets are chosen by analysing the learning curves of the prediction error^15^. This splitting procedure simulates the presence of future unseen data and it is realized ensuring that the age distribution of the samples in the two sets is the same. The second step consists in data standardization, meaning that each variable is transformed in order to have zero mean and unit variance. The third, and final, step consists in the use of the Elastic-Net model adopting a cross-validation scheme for parameter tuning^15^. In this work, we optimized the free parameters of the Elastic-Net model (***λ***, ***α***) in a grid-search cross-validation fashion, meaning that the best parameters are chosen from a two-dimensional grid as the pair yielding the lowest validation error estimated by 50 Monte-Carlo cross-validation resampling^27^ on the training set. The final weights vector is obtained refitting the model on the training set (which is the 75% of the whole dataset) using the optimal parameters. The performance of the achieved model is then assessed on the remaining test samples (i.e. the 25% of the full dataset).

Moreover, we have also explored the pairwise correlation across the collected measures with the Pearson Correlation Coefficient (*r*) (Eq. 3), which can be defined as:

3 $$r(x,z)=\frac{{{\rm{\Sigma }}}_{i=1}^{n}({x}_{i}-\bar{x})({z}_{i}-\bar{z})}{\sqrt{{{\rm{\Sigma }}}_{i=1}^{n}{({x}_{i}-\bar{x})}^{2}}\sqrt{{{\rm{\Sigma }}}_{i=1}^{n}{({z}_{i}-\bar{z})}^{2}}}$$

where *n* represents the number of samples and macrons above variables identify their empirical means. This measure can be seen as the ratio between the estimated covariance and the product of their empirical standard deviation. *r*(*x*, *z*) can be seen as a measure of linear correlation between two variables *x* and *z*. Pearson Correlation Coefficient values range from 1 (perfect positive linear correlation) and −1 (perfect negative linear correlation). A Pearson Correlation Coefficient value of 0 means that there is no linear correlation between the two variables.

## Supplementary information

Supplementary Figures

Supplementary Dataset
