# Supplementary material for: Discrete Changes in Glucose Metabolism Define Aging
Source: Sci Rep. 2019 Jul 17;9:10347. doi: 10.1038/s41598-019-46749-w (PMC6637183; doi:10.1038/s41598-019-46749-w)

Supplementary Information of the manuscript entitled:

DISCRETE CHANGES IN GLUCOSE METABOLISM DEFINE AGING.

Authors: Silvia Ravera, Marina Podestà, Federica Sabatini, Monica Dagnino, Daniela Cilloni, Samuele Fiorini, Annalisa Barla, Francesco Frassoni.

Figure 1 supplementary: Distribution of the population of MNC

Graph shows the distribution of the population of MNC isolated from PB of healthy population with age between 5 and 106 years. The population is divided by decades.

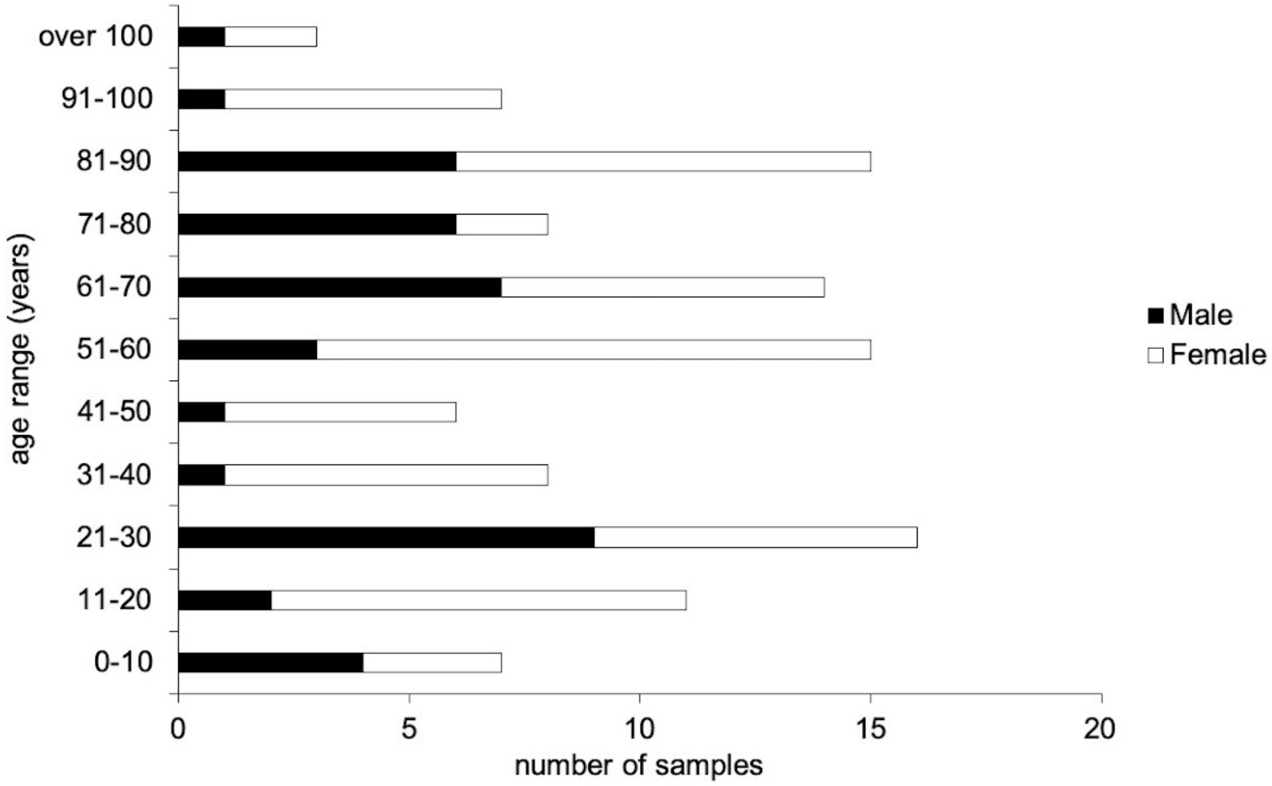

Figure 2 supplementary: ATP or AMP intracellular concentration in MNC

A and **B** show, respectively, the ATP or AMP intracellular concentration in MNC isolated from peripheral blood (PB), obtained from healthy population with an age between 5 and 106 years. ATP level remain stable during aging while AMP concentration increases. The population is divided by decades.

* or ** indicate, respectively, a significant difference for $p<0.05$ or $p<0.01$ between the marked decades and the previous decade.

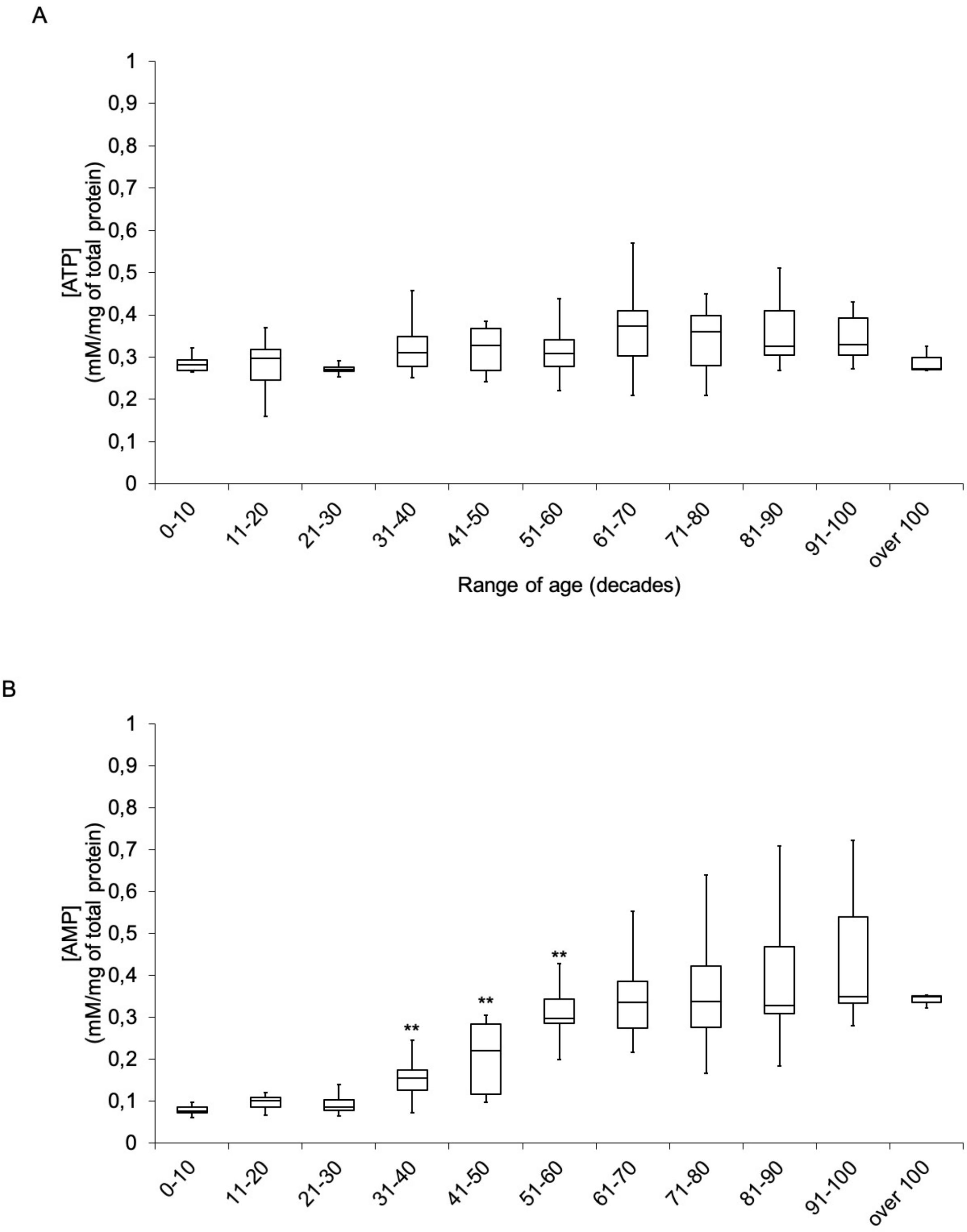

Supplement: Supplementary file 1 — Supplementary Figures [file 41598_2019_46749_MOESM1_ESM.pdf]
